# Facilitating Genetics Aware Clinical Decision Support: Putting the eMERGE Infrastructure into Practice

**DOI:** 10.1055/s-0041-1729981

**Published:** 2021-07-06

**Authors:** Casey Overby Taylor, Luke V. Rasmussen, Laura J. Rasmussen-Torvik, Cynthia A. Prows, David A. Dorr, Lipika Samal, Samuel Aronson

**Affiliations:** 1 Departments of Medicine and Biomedical Engineering, Johns Hopkins University, Baltimore, Maryland, United States; 2 Geisinger Health System, Genomic Medicine Institute, Danville, Pennsylvania, United States; 3 Department of Preventive Medicine, Northwestern University, Chicago, Illinois, United States; 4 Division of Human Genetics, Cincinnati Children*’*s Hospital Medical Center, Cincinnati, Ohio, United States; 5 Departments of Medical Informatics and Clinical Epidemiology and Medicine, Oregon Health & Science University, Portland, Oregon, United States; 6 Department of Medicine, Brigham and Women*’*s Hospital, Boston, Massachusetts, United States; 7 Partners Personalized Medicine, Partners HealthCare, Cambridge, Massachusetts, United States

**Keywords:** editorial, case report, genomics, pharmacogenomics, clinical decision support requirements, gathering information technology, infrastructure

## Abstract

This editorial provides context for a series of published case reports in ACI Open by summarizing activities and outputs of joint electronic health record integration and pharmacogenomics workgroups in the NIH-funded electronic Medical Records and Genomics (eMERGE) Network. A case report is a useful tool to describe the range of capabilities that an IT infrastructure or a particular technology must support. The activities we describe have informed infrastructure requirements used during eMERGE phase III, provided a venue to share experiences and ask questions among other eMERGE sites, summarized potential hazards that might be encountered for specific clinical decision support (CDS) implementation scenarios, and provided a simple framework that captured progress toward implementing CDS at eMERGE sites in a consistent format.

In our work as part of the National Institutes of Health National Human Genome Research Institute sponsored electronic Medical Records and Genomics (eMERGE) Network,^1,2^ we were presented the challenge of representing genetic test results in a standard format for use by clinical decision support (CDS). Ideally, genetic test results should exist in the electronic health record (EHR) as structured data to drive CDS for more personalized medication prescribing, diagnostic evaluation, and risk assessment.^3,4^ In addition, the genetic test result data and interpretations should be transmitted by using data representation standards.^5,6^ Few health care institutions, however, are accomplishing this objective.^7,8^ This is in part due to many health systems outsourcing genetic testing to external laboratories that do not transmit back machine-interpretable results. If we do not capture genetic test results in the EHR in a structured and standardized format, there are many downstream effects such as a lack of ability to manage updating results.^9,10^

To more fully develop, disseminate, and adopt a standard format for the transmission of genetic test results, real world use cases are needed to define the range of capabilities the standard must support. In this editorial, we provide an overview of our process to solicit requirements for producing, transmitting, and returning structured genetic test results. We also introduce an ACI Open case series that illustrates experiences of eMERGE clinical sites adopting standard formats for genetic test results as use cases and provides context for these use cases including some common challenges, takeaways, and future considerations for related efforts.

The eMERGE network has spanned more than a decade with three separate phases; each exploring the intersection of research discovery using EHR-linked biobanks, as well as the process of integrating genetic test results into clinical practice.^2,11,12^ In each phase, the network was composed of multiple sites, including academic medical centers, health systems, and genomic testing centers. Network members have representatives that participate in each of several workgroups that focus on specialized topics such as EHR integration, return of results, and pharmacogenomics.

During phase II of eMERGE, network participants identified the importance of returning clinical actionable variants implicated in disease processes.^11^ Building upon this, phase III focused on establishing and demonstrating mechanisms to return such results. This included infrastructure for the receipt, processing, and return of multigene sequencing data from two Clinical Laboratory Improvement Amendments-certified genomic sequencing centers.^1^ In addition, phase III aimed to explore mechanisms for return of pharmacogenomic (PGx) variants, further expanding the eMERGE-PGx project^13^ initiated in phase II of eMERGE.

Given the need to align infrastructure requirements between two genomic sequencing centers in eMERGE phase III, early in the project the EHR integration working group (EHRI WG) gathered feedback from participating sites. In particular, we conducted informal interviews of informatics and health information technology (HIT) contacts from each site on how they planned to use the reports and on what would be their process to transfer the data and reports from the laboratory into their clinical IT ecosystem. The findings from those interviews were presented as a part of a panel presentation at AMIA TBI 2016 titled “Practical Implementation of Genomic Sequencing in Healthcare Settings”^14^ and summarized.^3^ As a result of our findings, we enabled support for the needs of eMERGE sites in the final network infrastructure.^1^

In collaboration with local HIT teams, clinical sites defined additional implementation project requirements and infrastructure needs, including for CDS. For those sites participating in earlier phases of eMERGE, there were significant differences in the implementation plan in phase III that precluded reuse of existing infrastructure. This was driven by multiple factors, including the new study design, genomic test report format, mode of delivery of the reports (batch vs. individual report), changes in the list of genes and SNPs reported, changes in guidelines published by American College of Medical Genetics (ACMG) and Clinical Pharmacogenetics Implementation Consortium (CPIC), and, for some sites, changes in EHR vendors.

Given the variability in local implementation strategies, the EHRI WG established two mechanisms to continually share technical knowledge and lessons learned among network participants. First, we created a GitHub organization for workgroup members to share code used to process structured report data.^15^ This was due to our finding that the majority of eMERGE sites had plans to create a parser for those data. Second, we established a process for institutions to give updates during monthly EHRI WG calls to describe implementation progress. Site updates were based loosely on the Agile “daily scrum” format: what was done last month, what is being done this month, and what are road-blocks being encountered (if any)? The goal was to stick within 2 minutes for the update and then to provide flexibility for discussion among the group for another few minutes to address road-blocks.

Once reports and data were returned from the genomic sequencing centers to the network clinical sites, the group transitioned to sharing experiences with CDS for the return of genetic results. At the summer 2018 in-person meeting, the EHRI working group decided to dedicate time on monthly calls for a site to share in-depth descriptions of their local CDS efforts. This was an area that was in strong alignment with efforts being pursued in the eMERGE-PGx working group. As such, eMERGE-PGx workgroup members were invited to attend and present at monthly EHRI WG meetings. Subsequent in person meetings (held three times yearly) also included joint breakout sessions with the EHRI and PGx working groups.

One notable in-person meeting on June 21, 2019 in Seattle, WA specifically involved brainstorming potential hazards related to implementing CDS. We limited the scope of our hazards implementation considerations to alert-based CDS for (1) an update to a previously returned result due to new genetic variant knowledge and (2) a PGx alert in response to a drug order. We also considered two different architectural approaches for supporting genetic result management in EHR ecosystems: (1) the use of ancillary “omics systems^10^ and (2) EHR vendor supplied capabilities.” Hybrid scenarios were also considered. **►**Fig. 1 provides a summary of architectural approaches used by eMERGE sites. Published examples of both scenarios are also described elsewhere.^16,17^

Overall, we collected 25 potential hazards and identified 4 themes among them: inappropriate alert firing context (e.g., alert goes to the wrong clinician, alert does not reach all affected family members, etc.), technical issues (e.g., message lost during transmission between the laboratory and the clinic, mismatch between the format of the data result after an update, etc.), user experience problems (e.g., clinician is alerted and misinterprets the guidance, no disclosure of alert to the patient even though it is in the record, etc.), and knowledge maintenance (e.g., laboratory is no longer around to provide updates, discordant laboratory interpretations are not resolved). These findings can serve as a helpful starting point for more in depth hazards analysis exercises that involve identifying infrastructure specific hazards, classifying them based on severity and likelihood of occurrence, evaluating mitigations capable of reducing hazard likelihood and/or severity and finally determining if the overall application risk profile is acceptable. While this brainstorming exercise was helpful preparation to anticipate and mitigate challenges to CDS implementation, the monthly virtual workgroup meetings proved very useful to share experiences and lessons as implementation proceeded.

To classify and synthesize CDS implementations among eMERGE network sites, we produced a simple framework (**►**Fig. 2). The framework depicts three dimensions of user-system interactions with genetics aware CDS systems: (1) timing, (2) delivery, and (3) context. Over the course of the project, we continued to update responses from sites and added additional questions regarding the implementation of CDS for the return of results relevant to the American College of Medical Genetics and Genomics genes. At our final in person meeting for eMERGE phase III held in February 2020, we confirmed the current state of CDS implementation with EHRI WG and eMERGE-PGx working group members. A summary of findings is shown in **►**Fig. 1 and **►**Fig. 3.

Annually, all eMERGE sites met and presented to an outside expert scientific panel. Both the EHRI and PGx working groups often highlighted our lessons learned to the expert scientific panel. The panel praised both groups on communicating these lessons within the network, but also repeatedly urged both groups to find a way to disseminate more broadly. The panel felt strongly that communicating lessons learned beyond the network could help those from other health systems to simplify their approaches to implement genetics aware CDS in the EHR.

This ACI open case series includes submissions describing the individual experiences of several eMERGE phase II and III institutions and affiliate sites. The case reports published in this series illustrate some of the variability in how CDS was implemented among eMERGE institutions. There was a large amount of variation in the governance and operational implementation which highlights how prevalent these issues are in applied clinical informatics. In the article by Rasmussen et al, delays between planning, approval, and implementation caused confusion among participants. Prows et al discovered variability in the desire to see genetic information between adolescents and their guardians, leading to challenges in how information could be released. Overall, the complexities in sharing genetic information through EHRs require substantial collaboration and communication between various stakeholders, especially patients and families.

In summary, we have provided an overview of joint EHR integration and pharmacogenomics working group activities to provide context for a series of published case reports in ACI Open. Our combined activities have informed infrastructure requirements used during eMERGE phase III, provided a venue to share experiences and ask questions among other eMERGE sites, summarized potential hazards that might be encountered for specific CDS implementation scenarios, and provided a simple framework that captured progress toward implementing CDS at eMERGE sites in a consistent format. These approaches helped eMERGE sites to anticipate and mitigate challenges, avoid repeating similar mistakes, and to take advantage of approaches that worked for others. This commentary provides an overview of successes with establishing shared infrastructure for the return of genetic results and the final outcomes of implementing CDS during eMERGE phase III. This summary, however, by its nature, cannot not adequately represent the lessons learned from the unique experiences of eMERGE sites.

This ACI Open case series serves as a venue to provide a more in-depth view of implementation in away that illustrates variability in how CDS was implemented, the challenges encountered and best practices established for their needs. As recognized previously, such variability can lead to downstream barriers for multisite analyses.^19^ This series provides a way for the eMERGE network sites to convey critical lessons from implementing genetics with CDS into their EHR ecosystems. We believe that by communicating these lessons and best practices, we can inform more uniform CDS implementation strategies that leverage standards (e.g., SMART on FHIR,^20^ CDS Hooks,^21^ etc.) and facilitate easier implementation of genetic results with CDS into EHRs at other clinical sites.

## Figures and Tables

**Fig. 1:**
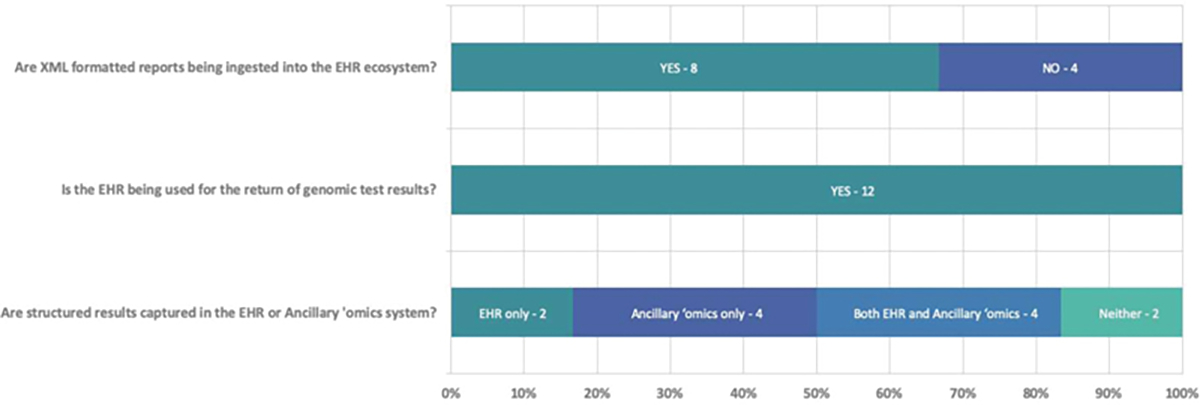
Summary of architectural approaches to capture and return genomic test results. This Figure summarizes responses to questions about the use of the structured reports and their integration into EHR and/or ancillary *‘*omics systems. In eMERGE phase III genomic test reports were provided to sites in both a PDF and structured (XML) format. Of note, some sites that participated in eMERGE-PGx, but that were not part of eMERGE phase III did not receive XML files. These data are from 12 eMERGE sites (Cincinnati Children*’*s Hospital Medical Center, Children*’*s Hospital of Philadelphia, Columbia University, Geisinger, Harvard University, Kaiser Permanente Washington with the University of Washington and the Fred Hutchinson Cancer Center, Marshfield Clinic, Mayo Clinic, Meharry Medical College, Mount Sinai, Northwestern University, Vanderbilt University Medical Center). eMERGE, electronic medical records and genomics.

**Fig. 2:**
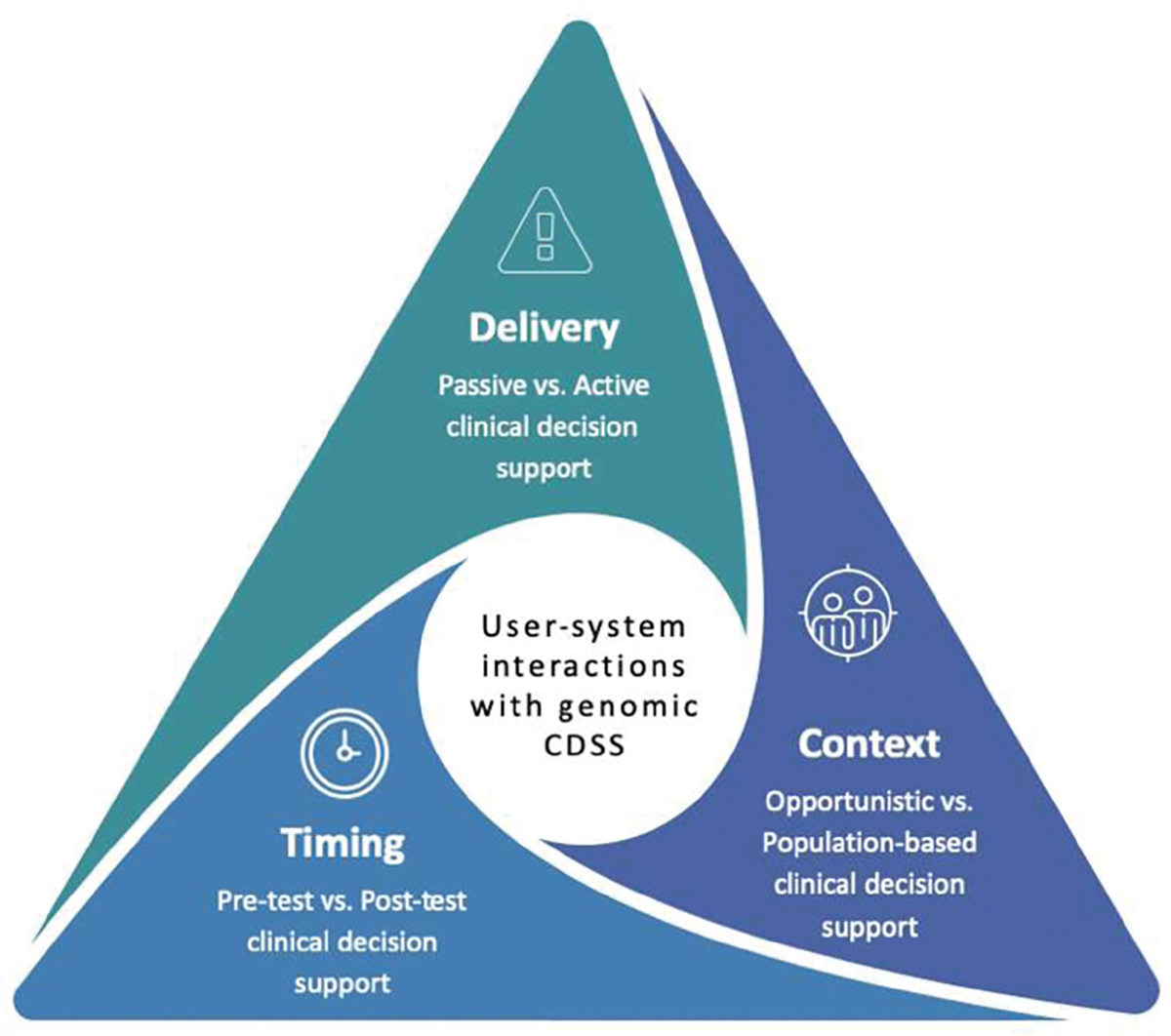
Three dimensions of user-system interactions for genomic CDS systems. The timing dimension included pretest and post-test CDS. Pretest CDS are presented prior to genomic test results being available, and post-test CDS provide guidance based on findings from existing genomic test results. The delivery dimension included passive and active CDS. Passive CDS uses a manual process to access information, for example, by clicking on a button. Active CDS uses an automated process to present information, for example, by displaying an alert message. The context dimension included opportunistic and population-based screening. Opportunistic screening CDS would offer patients secondary results related to conditions for which they have a low prior probability when they undergo sequencing for another purpose, for example, for research purposes. Population screening CDS would offer healthy individuals genomic sequencing as part of preventive health care.^17^ CDS, clinical decision support; eMERGE, electronic medical records and genomics.

**Fig. 3:**
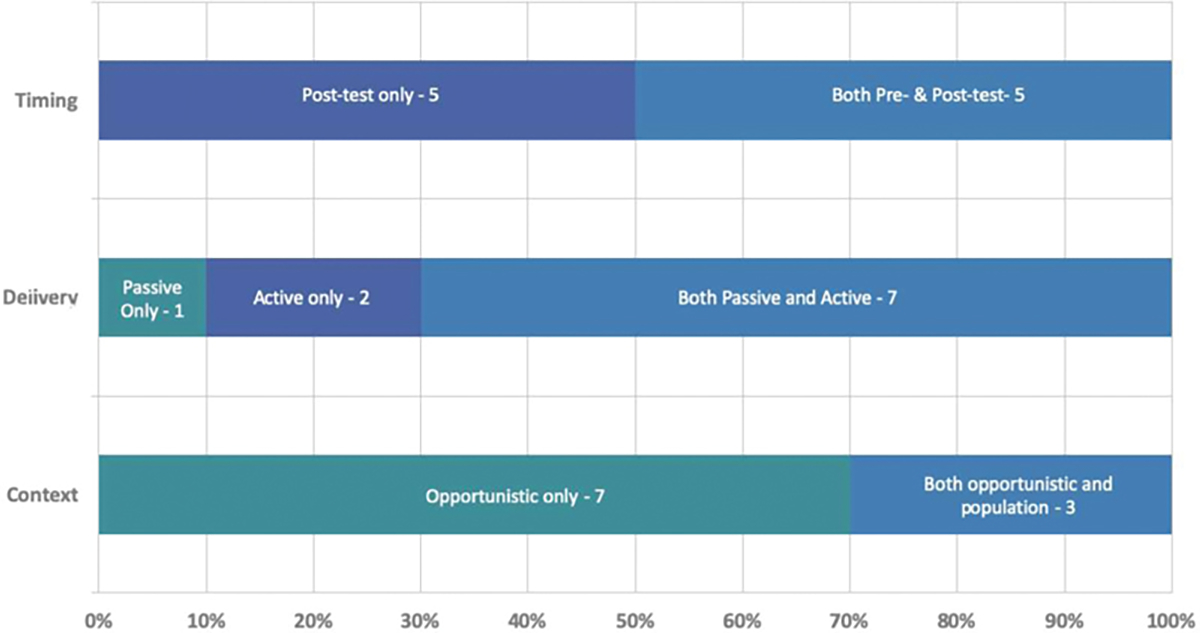
Summary of findings from survey of the three dimensions. These data are from 10 eMERGE sites (Cincinnati Children*’*s Hospital Medical Center, Children*’*s Hospital of Philadelphia, Columbia University, Geisinger, Harvard University, Kaiser Permanente Washington with the University of Washington and the Fred Hutchinson Cancer Center, Mayo Clinic, Mount Sinai, Northwestern University, Vanderbilt University Medical Center). Notably, all eMERGE sites provided opportunistic CDS, given the patients were all study participants that may receive secondary results. A subset of eMERGE sites also provided population-based CDS as part of institutional initiatives that were beyond the scope of this project. CDS, clinical decision support; eMERGE, electronic medical records and genomics.
